# Ventral Pallidum Neurons Are Necessary to Generalize and Express Fear-Related Responding in a Minimal Threat Setting

**DOI:** 10.1523/ENEURO.0124-24.2024

**Published:** 2024-11-26

**Authors:** Emma L. Russell, Michael A. McDannald

**Affiliations:** Department of Psychology & Neuroscience, Boston College, Chestnut Hill, Massachusetts 02467

**Keywords:** associative, conditioned suppression, fear generalization, probabilistic

## Abstract

Fear generalization is a hallmark of anxiety disorders. Experimentally, fear generalization can be difficult to dissociate from its counterpart, fear discrimination. Here, we use minimal threat learning procedures to reveal such a dissociation. We show that in Long–Evans rats, an auditory threat cue predicting footshock on 10% of trials produces a discriminated fear response that does not generalize to a neutral auditory cue. In contrast, even slightly higher footshock probabilities (30 and 20%) produce fear generalization. AAV-mediated, caspase-3 deletion of ventral pallidum neurons abolishes fear generalization and reduces threat cue responding during extinction. The ventral pallidum's contribution to fear generalization and extinction threat responding does not depend on inputs from the nucleus accumbens. The results demonstrate a minimal threat learning approach to dissociate fear discrimination from fear generalization and a novel role for the ventral pallidum in generalizing and expressing fear.

## Significance Statement

In the laboratory, healthy mice, rats, and people generalize fear responding to a neutral cue before showing fear discrimination. However, in the real world, fear generalization is not nearly as ubiquitous in healthy individuals. Here we show that in rats, minimal threat learning procedures manipulating footshock probability identify a boundary at which fear discrimination proceeds in the absence of fear generalization. We exploit this boundary to reveal a novel and essential role of the ventral pallidum in fear generalization.

## Introduction

The ability to predict and respond to environmental threats is essential to survival. When fear responding is confined to cues associated with threats, it is healthy and adaptive. However, fear responses that generalize to neutral cues, are maladaptive. Fear generalization is a hallmark of anxiety disorders (Cooper et al., 2022). Uncovering neural mechanisms for fear generalization is paramount to reducing the personal and societal burden of anxiety disorders.

A barrier to revealing neural mechanisms for fear generalization is a lack of behavioral procedures to distinguish fear generalization from its counterpart: fear discrimination (see Table 1 for definitions). This is because common laboratory approaches produce a behavioral pattern in which fear generalization necessarily precedes fear discrimination. For example, a common procedure uses a threat cue that predicts footshock (100% shock probability) and a neutral cue that does not (0% probability). In the laboratory, healthy people (Holt et al., 2014), intact rats (McDannald, 2010; Foilb et al., 2018), and intact mice (Jo et al., 2018) initially acquire threat-like responses to a neutral cue. As the procedure continues, subjects retain responding to the threat cue and reduce threat-like responding to the neutral cue.

**Table 1.** Definitions for terms

Term	Definition
Fear learning	Acquiring a behavioral response to a threat cue paired with shock
Fear discrimination	Differential responding to a threat cue (paired with shock) and a neutral cue (not paired with shock)
Fear generalization	Threat-like responding to a neutral cue

Operationalized definitions for fear learning, fear discrimination, and fear generalization.

This pattern may lead to the assumption that fear generalization and fear discrimination represent extremes of a single process. This view has significant implications for the neural mechanisms underlying fear generalization. If fear generalization and fear discrimination are extremes of a single process, both are likely to be the product of the same neural mechanisms. However, if fear generalization and fear discrimination are distinguishable processes, each is likely to be supported by unique neural mechanisms.

The basolateral amygdala plays a critical role in acquiring responding to threat cues (LeDoux et al., 1988; Helmstetter, 1992; Maren, 1999; Goosens and Maren, 2001; Lee et al., 2001, 2005; Nader et al., 2001; Choi and Brown, 2003; Gale et al., 2004; Koo et al., 2004; Anglada-Figueroa and Quirk, 2005; Petrovich et al., 2009; McDannald, 2010; Liu et al., 2022; Williams-Spooner et al., 2022). However, it is now recognized that the amygdala does not work alone to acquire and express fear. The second goal of this study was to reveal possible roles of the ventral pallidum in generalizing and expressing fear responding in this minimal threat setting. Why the ventral pallidum? The ventral pallidum is anatomically positioned to modulate fear responding. The ventral pallidum is a forebrain region with direct and extensive projections to the basolateral amygdala (Haber et al., 1985; Root et al., 2015). Furthermore, the ventral pallidum contains signals that are suitable to guide threat responding and utilize error to modify cue–shock associations. In reward settings (Smith et al., 2009), the ventral pallidum has been shown to signal a reward value (Ottenheimer et al., 2018; Stephenson-Jones, 2019), as well as reward prediction error (Ottenheimer et al., 2020). The ventral pallidum reward value (Tachibana and Hikosaka, 2012) and reward prediction error signals (Ottenheimer et al., 2020) incorporate trial history into their respective computations. In aversive settings, ventral pallidum neurons signal relative threat value (Kaplan et al., 2020; Stephenson-Jones et al., 2020; Moaddab et al., 2021).

Beyond the amygdala, the ventral pallidum is reciprocally connected with the nucleus accumbens core (Haber et al., 1985). Like the ventral pallidum itself, this pathway is consistently implicated in reward settings (Pardo-Garcia et al., 2019). However, recent evidence indicates roles of this same pathway in fear and anxiety. Optogenetic excitation of D2R accumbens neurons projecting to the ventral pallidum promotes anxiety-like behavior (Correia et al., 2023). The accumbens to the ventral pallidum pathway may play a more general role in eliciting and organizing fear and anxiety behavior.

The goals of the present experiments were threefold. In Experiment 1, we sought to develop behavioral procedures that dissociate fear discrimination from fear generalization. To do this, we arranged for rats to undergo minimal threat learning in which a neutral cue never predicted footshock, while a threat cue predicted a low probability of footshock (30, 20, or 10%). We monitored responding during 10 learning sessions and then a single extinction session. In Experiment 2, we asked if there is any role of the ventral pallidum in fear generalization, fear discrimination, or the expression of fear-related responding during extinction testing. Rats had ventral pallidum neurons bilaterally deleted using an intersectional AAV approach (Yang et al., 2013; Tervo et al., 2016). Control rats had ventral pallidum neurons left intact. Rats were then assessed in minimal threat learning procedures (30 or 10% threat probability). Finally, we asked if any of these behavioral processes depended specifically on the accumbens to the ventral pallidum pathway. Nucleus accumbens neurons projecting to the ventral pallidum were either deleted using an intersectional AAV approach or left intact. Pathway-deleted and intact rats were then assessed in all aspects of the 30% minimal threat learning procedure.

## Materials and Methods

### Subjects

Subjects were 94 Long–Evans rats (47 females) arriving from Charles River Laboratories on Postnatal Day 55, weighing 196–298 g (Experiment 1, 47 total, 23 females; Experiment 2, 23 total, 11 females; and Experiment 3, 24 total, 12 females). Upon arrival, rats were single-housed and placed on a 12 h light cycle (lights off at 6:00 P.M.). Rats acclimated to the facility with *ad libitum* access to water and chow (18% Protein Rodent Diet 2018, Harlan Teklad Global Diets) for at least 2 weeks before behavioral testing or surgery. All experiments were performed in accordance with the NIH guidelines regarding the care and use of rats for experimental procedures.

### Surgery

Aseptic, stereotaxic surgery was performed under isoflurane anesthesia (1–5% in oxygen) for Experiments 2 and 3. Carprofen (5 mg/kg, s.c.), lactated ringer's solution (5 ml, s.c.), and 2% lidocaine (∼1.5 ml, s.c.) were administered preoperatively.

#### Experiment 1

No surgery was performed.

#### Experiment 2

The cranium was exposed via midline incision. Two holes were drilled above the ventral pallidum at sex-specific coordinates: AP −0.12 mm and ML ±2.05 mm (11 females) and AP −0.08 mm and ML ±2.05 mm (12 males). Rats received bilateral, 0.3 µl infusions of eSyn-iCre-T2A-eGFP-WPRE (rAAV2-retro) at DV −8.00 mm (female) or DV −8.10 mm (male). RAAV2-retro inserts cre recombinase into local ventral pallidum neurons, as well as to neurons projecting to the ventral pallidum. Casp3^VP^ rats received bilateral 1.0 µl infusions of AAV-flex-taCasp3-TEVp (caspase-3 induces apoptosis in cre-containing neurons) at the rAAV2-retro coordinates. Control rats received bilateral 1.0 µl infusions of AAV-hSyn-mCherry at the rAAV2-retro coordinates. The dual viral approach meant ventral pallidum neurons would be deleted in Casp3^VP^ rats but left intact in control rats.

Infusions were delivered via 2 µl syringes (Hamilton, Neuros) controlled by a microsyringe pump (World Precision Instruments, UMP3-2). The syringe was raised 0.1 mm 1 min after the completion of each infusion and then left in place for 5 min to encourage delivery to the target site. Syringes were rinsed with saline between infusions.

#### Experiment 3

Bilateral eSyn-iCre-T2A-eGFP-WPRE infusions were performed as in Experiment 2. Now, two additional holes were drilled above the nucleus accumbens core: AP +1.90 mm and ML ±1.80 mm (*n* = 24; 12 females). Casp3^NAcc→VP^ rats (*n* = 12; six females) received bilateral 1.0 µl infusions of AAV-flex-taCasp2-TEVp into the nucleus accumbens core. Control rats (*n* = 12; six female) received bilateral 1.0 µl infusions of AAV-EF1a-DIO-mCherry (*n* = 12). The dual viral approach meant ventral pallidum-projecting nucleus accumbens neurons would be deleted in Casp3^NAcc→VP^ rats but left intact in control rats. A complete breakdown of rats by sex, threat probability condition, and surgical group is provided in Table 2.

**Table 2.** Rat numbers by group, probability, and sex

Group	Probability	Sex	*n*
Experiment 1			
	30%	Female	8
	30%	Male	8
	20%	Female	8
	20%	Male	8
	10%	Female	7
	10%	Male	8
Experiment 2			
Control^VP^	30%	Female	3
Control^VP^	30%	Male	3
Control^VP^	10%	Female	3
Control^VP^	10%	Male	3
Casp3^VP^	30%	Female	3
Casp3^VP^	30%	Male	3
Casp3^VP^	10%	Female	2
Casp3^VP^	10%	Male	3
Experiment 3			
Control^NAc→VP^	30%	Female	6
Control^NAc→VP^	30%	Male	6
Casp3^NAc→VP^	30%	Female	6
Casp3^NAc→VP^	30%	Male	6

The number of rats in each surgical group and footshock probability condition are shown by sex.

### Postoperative procedures

Rats received Rimadyl (Bio-Serv MD150-2, 2 mg/tablet) for postoperative analgesia for 2 d postsurgery. At least 4 weeks passed between surgery and footshock sessions to permit caspase-3–mediated neuronal deletion.

### Behavior apparatus

**Figure 1.**
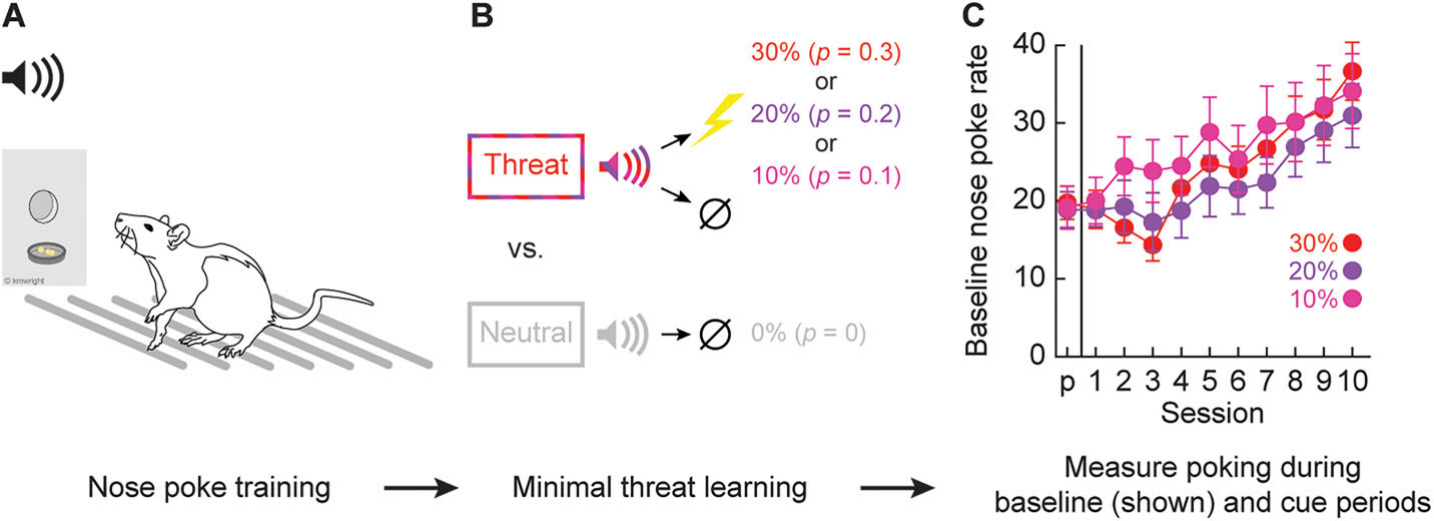
Experimental design. ***A***, Rats nose poke for food. Independently, cues can be played through overhead speakers, and shocks can be delivered through metal floor bars. ***B***, Minimal threat learning consisted of 10 s auditory cues predicting unique footshock probabilities: threat (red, *p *= 0.3; purple, *p *= 0.2; and pink, *p *= 0.1) and neutral (gray; *p* = 0). Footshock was 2 s following cue offset on trials it was presented. ***C***, The Mean ± SEM baseline nose poke rate during the cue pre-exposure session (*p*) and the 10 sessions of shock presentation (1–10) is shown. Baseline nose poke rates are shown because they are used to calculate cue suppression ratio.

The apparatus for behavioral testing consisted of eight individual chambers with aluminum front and back walls, clear acrylic sides and top, and a grid floor. Each floor bar was electrically connected to an aversive shock generator (Med Associates). An external food cup and a central port equipped with infrared photocells were present on one wall. Auditory stimuli were generated with an Arduino-based device or a WAV trigger-based device and presented through two speakers mounted on the ceiling (Fig. 1*A*). Each experimental chamber was enclosed in a sound-attenuating shell.

### Pellet exposure and nose poke shaping

Rats were food-restricted and specifically fed to maintain their body weight throughout behavioral testing. Each rat was given 4 g of experimental pellets (Bio-Serv Dustless Precision Pellets, 45 mg, Rodent Purified Diet, F0021) in their home cage in order to overcome neophobia for 2 d. Next, the central port was removed from the experimental chamber, and rats received a 30 min session in which one pellet was delivered every minute. Following the pellet session, the central port was returned to the experimental chamber for the remainder of the behavioral testing. Each rat was first shaped to nose poke in the central port for experimental pellet delivery using a fixed ratio schedule in which one nose poke into the port yielded one pellet. Shaping sessions lasted 30 min or until ∼15 nose pokes were completed. Each rat then received five sessions during which nose pokes into the port were reinforced on a variable interval schedule. Session 1 used a variable interval of 30 s schedule (poking into the port was reinforced every 30 s on average). All remaining sessions used a variable interval of 60 s schedule. For the remainder of behavioral testing, nose pokes were reinforced on a variable interval of 60 s schedule independent of cue and shock presentation. A variable schedule, rather than a ratio schedule, was used to encourage consistent nose poking across the entire session.

### Cue pre-exposure

Each rat was pre-exposed to the two cues to be used in the minimal threat learning procedure. Auditory cues were 10 s in duration and consisted of repeating, 500 ms motifs of broadband click or trumpet. A 49 min session consisted of 10 presentations of each cue (20 total presentations) with a mean intertrial interval (ITI) of 2 min. The cue presentation order was randomly determined by the behavioral program and differed for each rat, each session.

### Minimal threat learning

#### Experiment 1

Each rat received 10, 49 min minimal threat learning sessions consisting of 20 trials, with a mean ITI of 2 min. Rats were divided into three groups. Each rat received 10 threat cue presentations. There were three probability conditions with the threat cue being followed by footshock on 10% (*n* = 15), 20% (*n* = 16) or 30% (*n* = 16) of trials (Fig. 1*B*). Each rat also received 10 presentations of a neutral cue that was never followed by footshock. Auditory identities of the threat and neutral cues were counterbalanced across rats. Footshock (0.5 s, 0.5 mA) was administered 2 s following shock termination on trials in which it was given. The order of threat and neutral cue presentation was randomly determined by the behavioral program and differed for each rat, each session.

#### Experiment 2

The behavioral procedure is as in Experiment 1, but only the 10 and 30% threat probabilities were used.

#### Experiment 3

The behavioral procedure is as in Experiment 1, but only the 30% threat probability was used.

### Calculating suppression ratio

Behavioral data were acquired using Med Associates, Med-PC IV software. Raw data were processed in MATLAB to extract time stamps for nose poke initiation and cue onset. Fear-related behavior was measured with suppression ratio using nose poke rates from the baseline and cue periods (poke/min). The baseline nose poke rate came from the 20 s precue period. The cue nose poke rate came from the 10 s cue period. The suppression ratio was calculated as follows: (baseline poke rate − cue poke rate) / (baseline poke rate + cue poke rate). A suppression ratio of “1” indicated complete suppression of nose poking during cue presentation relative to the baseline. A suppression ratio of “0” indicated equivalent nose poke rates during the baseline and cue presentation, meaning no suppression of nose poking. Gradations in suppression ratio between 1 and 0 indicated intermediate levels of nose poke suppression during cue presentation relative to the baseline. Negative suppression ratios indicated increased nose poke rates during cue presentation relative to the baseline.

Note: suppression of reward seeking is not “fear.” Fear, referring to behavioral output, is a collection of many overt and autonomic responses elicited by a shock-paired cue (Davis, 1992; McDannald, 2023). Suppression of reward seeking is one behavioral property of a footshock-associated cue.

### Histology

Upon the conclusion of behavioral testing for Experiments 2 and 3, rats were anesthetized with an overdose of isoflurane and perfused intracardially with 0.9% biological saline and then 10% neutral buffered formalin. Brains were extracted and postfixed in 10% neutral buffered formalin for 24 h and then stored in 4% formalin and 10% sucrose for an additional 24 h. Forty micrometer sections were collected on a sliding microtome and stored in cryoprotectant until mounting.

The tissues were collected around the bregma levels +0.36, 0.00, and −0.36 (Experiment 2) and bregma levels 0.00 and +1.68 (Experiment 3) and then washed with 0.2 M potassium phosphate-buffered solution (3 × 5 min). The tissues were mounted on slides and left to dry overnight in a dark area. Dry slides were coverslipped with Vectashield Hardset mounting media (Vector Labs). Sections were imaged for green fluorescent protein (GFP) within 3 weeks of processing. A subset of sections were processed with immunohistochemistry for substance P (primary antibody, rabbit anti-substance P, 1:100, ImmunoStar; secondary antibody, Alexa Fluor 594 donkey anti-rabbit, Jackson ImmunoResearch Laboratories) and NeuroTrace (1:200, Thermo Fisher Scientific) to determine overlap of GFP expression with the ventral pallidum as defined by the location of substance P fibers.

### Image acquisition and GFP neuron counting

Images were captured using a light microscope (Axio Imager Z2, Carl Zeiss). GFP neuron counting was performed with Fiji (Schindelin et al., 2012). Color images were converted to grayscale and then thresholded to optimize the signal to the background ratio. A Gaussian blur was applied, the image was converted to binary, and then the number of GFP particles was counted. Two ventral pallidum sections (∼0.00 bregma, left hemisphere + right hemisphere) were quantified for Experiments 2 and 3. Two ventral pallidum sections (bregma 0.00, left hemisphere + right hemisphere) and two nucleus accumbens sections (bregma +1.68, left hemisphere + right hemisphere) were quantified for Experiment 3. Note: the software and lens for our microscope were updated between Experiments 2 and 3. This led to overall differences in fluorescence levels between the two experiments.

### Statistical analyses

Nose poke and suppression ratio data were analyzed with analysis of variance (ANOVA); *p *< 0.05 was considered significant. Within-subject, post hoc comparisons were performed using 95% bootstrap confidence intervals (95% BCIs). For 95% BCIs, observing confidence intervals that did not contain zero indicated differences between conditions. The 95% BCIs were preferred because they do not assume normality and avoid Type 1 error issues associated with multiple *t* tests. Between-subject, post hoc comparisons were performed using independent sample *t* tests; *p *< 0.05 was considered significant.

## Results

### Experiment 1

#### Baseline nose poke rates

Rats were mildly food deprived and trained to nose poke for a food reward. Poking was reinforced through minimal threat learning. Baseline nose poke rates increased over the 11 sessions, with increases greatest observed for males and rats in the 10% probability condition (Fig. 1*C*). ANOVA for the baseline nose poke rate [between-subject factors, sex (female and male) and probability (10, 20 or 30%); within-subject factors, session (11, pre-exposure through Session 10)] found significant main effects of session (*F*_(10,410) _= 36.03; *p* = 2.68 × 10^−50^) and sex (*F*_(1,41) _= 23.80; *p* = 1.70 × 10^−5^). Additionally, ANOVA found significant interactions for session × sex (*F*_(10,410) _= 5.03; *p* = 6.46 × 10^−7^) and probability × session (*F*_(20,410) _= 1.95; *p* = 0.009). Female rats had lower mean baseline nose poke rates than males across testing (*t*_45 _= 5.22; *p *= 4.00 × 10^−6^).

#### Minimal threat learning

**Figure 2.**
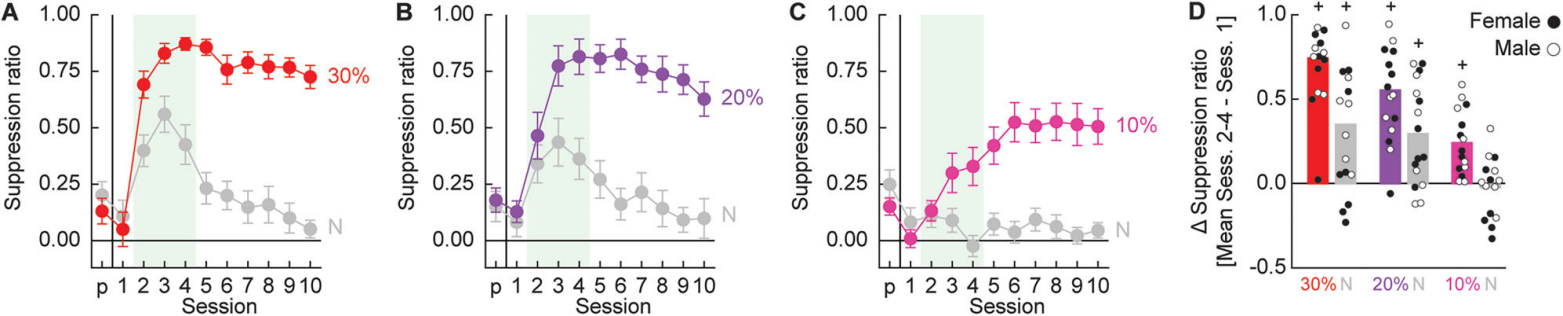
Responding during minimal threat learning. Mean ± SEM suppression ratios for threat (red, *p *= 0.3; purple, *p *= 0.2; and pink, *p *= 0.1) and neutral (gray, *p* = 0) from pre-exposure (*p*) through the 10 minimal threat learning sessions (1–10) are shown for rats in the (***A***) 30%, (***B***) 20%, and (***C***) 10% probability conditions. ***D***, Group Mean (bars) and individual (female, filled; male, open) change in suppression ratio from Session 1 to Sessions 2–4 (Mean) are shown. The ^+^95% BCI does not contain zero.

Nose poke suppression to the threat cue (30, 20, or 10%) and the neutral cue (0%) was measured over the 11 sessions. Discrimination emerged for rats in each probability condition (Fig. 2*A*–*C*). Generalization of suppression to the neutral cue was observed in the 30 and 20% probability conditions, but not the 10% condition. Demonstrating discrimination, ANOVA [between-subject factors, sex (female and male) and probability (10, 20 or 30%); within-subject factors, cue (threat vs neutral) and session (11, pre-exposure through session 10)] found significant main effects of cue (*F*_(1,41) _= 255.09; *p* = 3.32 × 10^−19^), session (*F*_(10,410) _= 27.61; *p* = 3.03 × 10^−40^), a significant cue × session interaction (*F*_(10,410) _= 41.69; *p* = 1.76 × 10^−56^), and a significant cue × probability interaction (*F*_(2.41) _= 3.74; *p* = 0.032). The threat cue elicited higher levels of suppression than the neutral cue, with the difference most apparent for 30 and 20% probability conditions during the later sessions.

ANOVA also found a significant main effect of probability (*F*_(2,41) _= 16.43; *p* = 6.00 × 10^−6^) and a significant session × probability interaction (*F*_(20,410) _= 5.43; *p* = 2.51 × 10^−12^). For the 30 and 20% conditions, the rapid increase in threat cue suppression ratios from Session 1 to Sessions 2–4 was also observed to the neutral cue. The 95% BCIs for differential suppression ratios [mean (M) Sessions 2–4 − Session 1] did not contain zero for any cue/group (Fig. 2*D*). For the 10% condition, the rapid increase in threat cue suppression ratio from Session 1 to Sessions 2–4 (Mean, 0.25; 95% CI [(lower bound) 0.15, (upper bound) 0.34]) was not observed to the neutral cue (M, −0.03; 95% CI [−0.11, 0.07]).

#### Influence of biological sex

The same ANOVA found a significant main effect of sex (*F*_(1,41) _= 8.58; *p* = 0.006), a significant session × sex interaction (*F*_(10,410) _= 2.32; *p* = 0.012), and a significant session × sex × probability interaction (*F*_(20,410) _= 2.30; *p* = 0.001). Females showed higher overall levels of suppression, particularly during the later sessions. No significant sex × cue interactions were observed.

#### Extinction test

**Figure 3.**
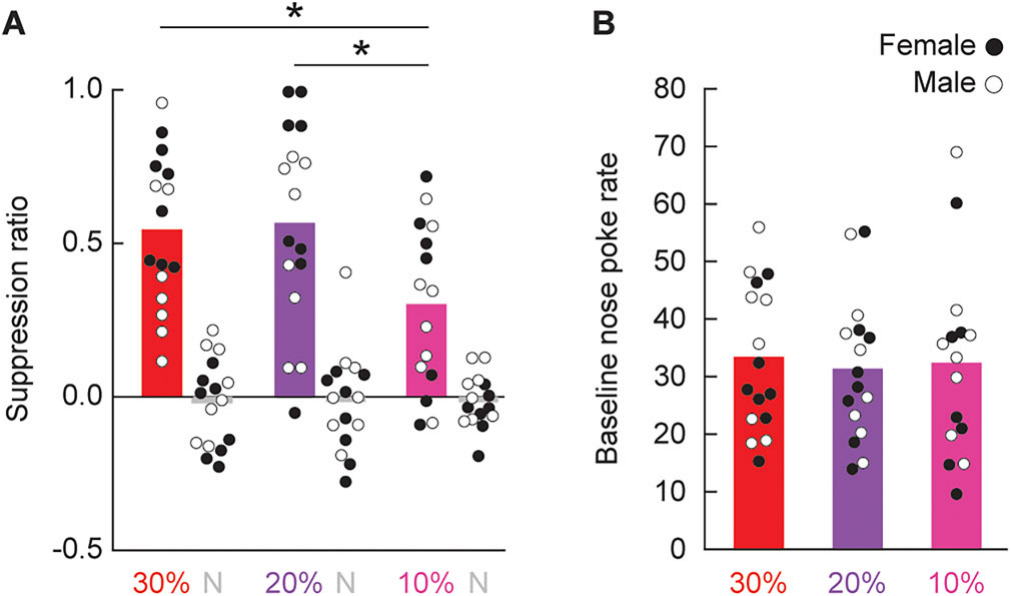
Responding during extinction test. ***A***, Group Mean (bars) and individual (female, filled; male, open) suppression ratios for threat (red, *p *= 0.3; purple, *p *= 0.2; and pink, *p *= 0.1) and neutral (gray, *p *= 0) from the extinction test are shown. ***B***, Group Mean and individual baseline nose poke rates are shown (color as in ***A***). *Independent samples *t* test, *p *< 0.05.

Extinction testing revealed discrimination and partial sensitivity to the threat probability manipulations (Fig. 3*A*). Rats in each condition showed greater suppression to the threat cue compared with that to the neutral cue. Rats in the 30 and 20% threat probability conditions showed greater threat cue suppression than rats in the 10% condition. Confirming discrimination, ANOVA [between-subject factors, sex (female and male) and probability (10, 20 or 30%); within-subject factor, cue (threat vs neutral)] found a significant main effect of cue (*F*_(1,41) _= 120.77; *p* = 8.5662 × 10^−14^). The 95% BCIs confirmed differential responding to threat and neutral cues by rats in the 30% (M, 0.56, 95% CI [0.41, 0.71]), 20% (M, 0.58, 95% CI [0.41, 0.74]), and 10% conditions (M, 0.31; 95% CI [0.18, 0.44]). Revealing partial sensitivity to the threat probability manipulations, ANOVA found a significant probability × cue interaction (*F*_(2,41) _= 3.51; *p* = 0.039), as well as a significant main effect of probability (*F*_(2,41) _= 3.96; *p* = 0.027). Between-subject *t* tests confirmed higher threat cue suppression ratios in the 30 versus 10% conditions (*t*_29 _= 2.60; *p *= 0.014) and in the 20 versus 10% conditions (*t*_29 _= 2.43; *p *= 0.021). Neutral cue suppression ratios did not differ between any groups (all *t* < 0.1; *p *> 0.9).

Female rats showed greater discrimination that was driven by higher suppression ratios to the threat cue. In support, ANOVA revealed a significant main effect of sex (*F*_(1,41) _= 14.69; *p* = 4.27 × 10^−4^) and a significant cue × sex interaction (*F*_(1.41) _= 6.91; *p* = 0.012). Group differences in suppression ratio could not be attributed to differences in baseline nose poking. All groups showed equivalent baseline nose poke rates during the extinction test (all *t* < 0.46; *p *> 0.6; Fig. 3*B*).

### Experiment 2

Experiment 1 revealed that a 30% threat cue produces discrimination plus generalization, while a 10% threat cue produces discrimination without generalization. Experiment 2 combined caspase-mediated neuronal deletion with the 30 and 10% minimal threat learning procedures to examine possible roles of the ventral pallidum in discrimination, generalization, and the expression of learning. If the ventral pallidum specifically contributes to generalization, then neuronal deletion should selectively abolish responding to the neutral cue in the 30% condition. If the ventral pallidum is necessary for discrimination, then differential responding to the threat cue and neutral cue should be impaired in both the 30 and 10% conditions. If the ventral pallidum is necessary for the expression of minimal threat learning, then threat cue responding should be diminished during the extinction test.

#### Histology

**Figure 4.**
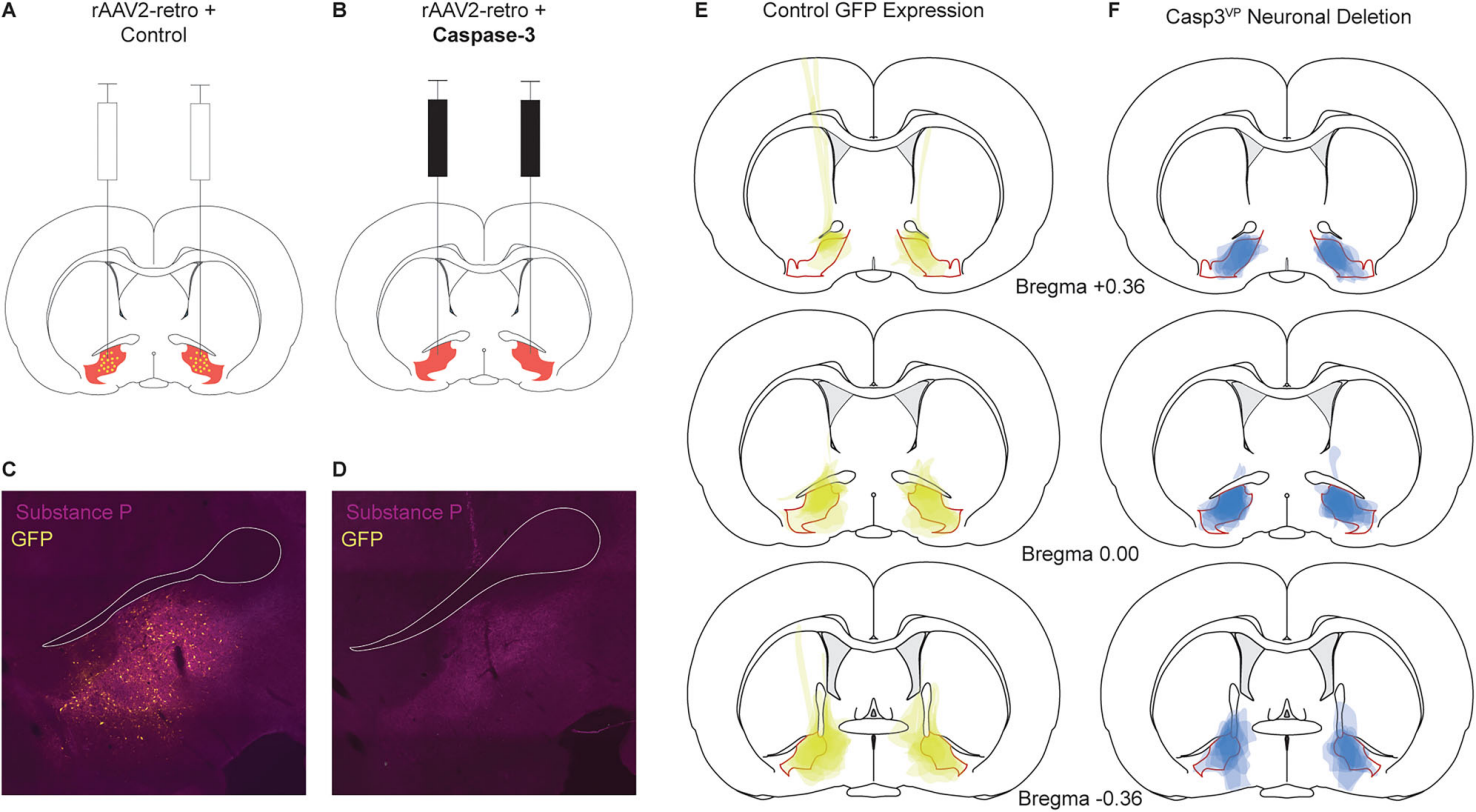
Dual viral approach and GFP/deletion mapping. ***A***, Control rats received bilateral rAAV2-retro and mCherry infusions into the ventral pallidum. ***B***, Casp3^VP^ rats received bilateral rAAV2-retro and cre-dependent caspase-3 into the ventral pallidum. The ventral pallidum indicated in red. Representative fluorescent images of subcommissural ventral pallidum are shown for (***C***) controls and for (***D***) Casp3^VP^ rats (substance P, magenta; GFP, yellow). ***E***, Individual control rat GFP expression (green–yellow) was mapped at bregma levels +0.36, 0.00, and −0.36, made transparent then overlayed. Areas of darker green–yellow indicate areas of more consistent GFP expression. ***F***, Individual Casp3^VP^ rat neuronal deletion (blue) was mapped at bregma levels +0.36, 0.00, and −0.36, made transparent then overlayed. Areas of darker blue indicate areas of more consistent neuronal deletion. The ventral pallidum border is outlined in red for both ***E*** and ***F***. Representative NeuroTrace images from a control and Casp3^VP^ rat are shown in Extended Data Figure 4-1.

Control^VP^ rats received bilateral rAAV2-retro and mCherry infusions into the ventral pallidum (Fig. 4*A*). Casp3^VP^ rats received bilateral rAAV2-retro and cre-dependent caspase-3 (Fig. 4*B*). Subcommissural GFP fluorescence was bilaterally quantified at bregma 0.00 for each rat (Fig. 4*C*,*D*). Consistent with the known property of rAAV2-retro to locally transfect cell bodies (Tervo et al., 2016), Control^VP^ rats showed high levels of GFP fluorescence and GFP + soma were apparent. Consistent with caspase-3–mediated deletion of ventral pallidum neurons, little or no GFP fluorescence was observed in Casp3^VP^ rats. GFP mapping (Fig. 4*E*) revealed viral expression throughout the anterior–posterior extent of the ventral pallidum. Deletion mapping (Fig. 4*F*) using NeuroTrace (Extended Data Fig. 4-1) revealed corresponding and nearly complete neuronal death at the same ventral pallidum levels. GFP expression and neuronal deletion were most focal at anterior and middle levels and then spread slightly beyond the bounds of the ventral pallidum in the posterior levels.

10.1523/ENEURO.0124-24.2024.f4-1 Figure 4-1 Representative NeuroTrace Images from Experiment 2 **(A)** A coronal section from a control rat containing the ventral pallidum is stained with NeuroTrace. The anterior commissure (AC) is outline in white. Sub-commissural ventral pallidum neurons are intact. **(B)** A coronal section from a caspase-3 rat containing the ventral pallidum is stained with NeuroTrace. The anterior commissure (AC) is outlined in white. Sub-commissural ventral pallidum neurons are deleted with little or no evidence of remaining neurons. Download Figure 4-1, TIF file.

**Figure 5.**
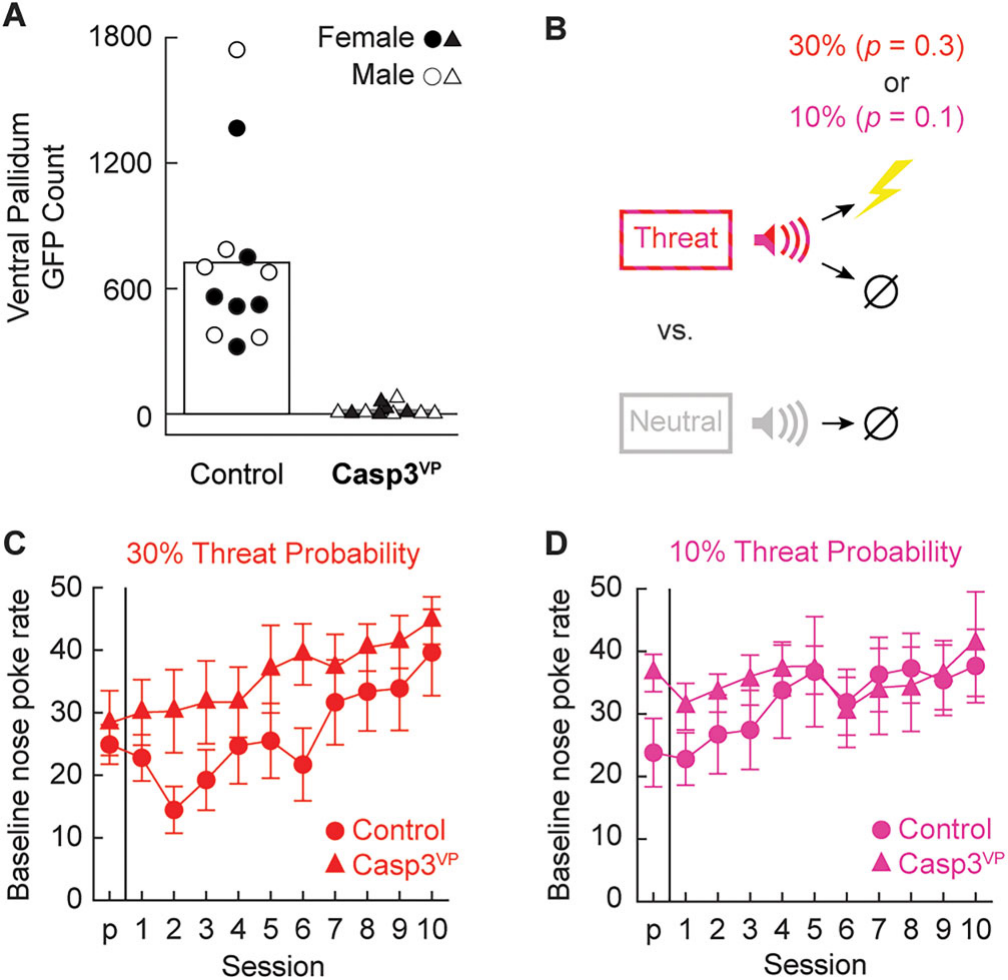
Quantifying GFP, experimental design, and baseline nose poke rate. ***A***, Group Mean (bars) and individual (female, filled; male, open) ventral pallidum neuron number is shown for control (circles) and Casp3^VP^ rats (triangles). ***B***, Minimal threat learning procedure consisted of 10 s auditory cues predicting unique footshock probabilities: threat (red, *p *= 0.3, and pink, *p *= 0.1) and neutral (gray, *p* = 0). The Mean ± SEM baseline nose poke rate from pre-exposure (*p*) through the 10 minimal threat learning sessions (1–10) is shown for control (circles) and Casp3^VP^ rats (triangles) in the (***C***) 30% (red) and (***D***) 10% (pink) probability conditions.

Independent samples *t* test found significantly higher ventral pallidum GFP counts in Control^VP^ (Mean, 724.33) compared with Casp3^VP^ rats (Mean, 17.82; *t*_(21) _= 5.51; *p *= 1.8 × 10^−5^; Fig. 5*A*). GFP expression did not differ between females and males nor between the 30 and 10% threat probability conditions (both *t* < 0.20; *p *> 0.80).

The combination of the rAAV2-retro construct and the camera lens/software settings is made for intense fluorescent signals. The GFP fluorescent signals were, in some sections, so intense it was difficult to discern individual neurons. To distinguish neurons, we applied a watershed transform. This may have inflated the overall neuron number. Identical settings were used for the control and Casp3^VP^ imaging. Observing little or no GFP expression in Casp3 sections in these intense fluorescent conditions makes us confident deletion was not only successful, but nearly absolute.

#### Baseline nose poke rates

Over the 11 sessions of minimal threat learning (Fig. 5*B*), the baseline nose poke rate increased for all groups (Fig. 5*C,D*). Female Casp3^VP^ rats poked at higher rates than female Control^VP^ rats. ANOVA for the baseline nose poke rate [between-subject factors, sex (female and male), group (Control^VP^ and Casp^VP^), and probability (10 or 30%); within-subject factors, session (11, pre-exposure through Session 10)] found a significant main effect of session (*F*_(10,150) _= 9.02; *p *= 1.51 × 10^−11^) and a significant sex × group interaction (*F*_(1,15) _= 8.68; *p *= 0.010).

#### Minimal threat learning

**Figure 6.**
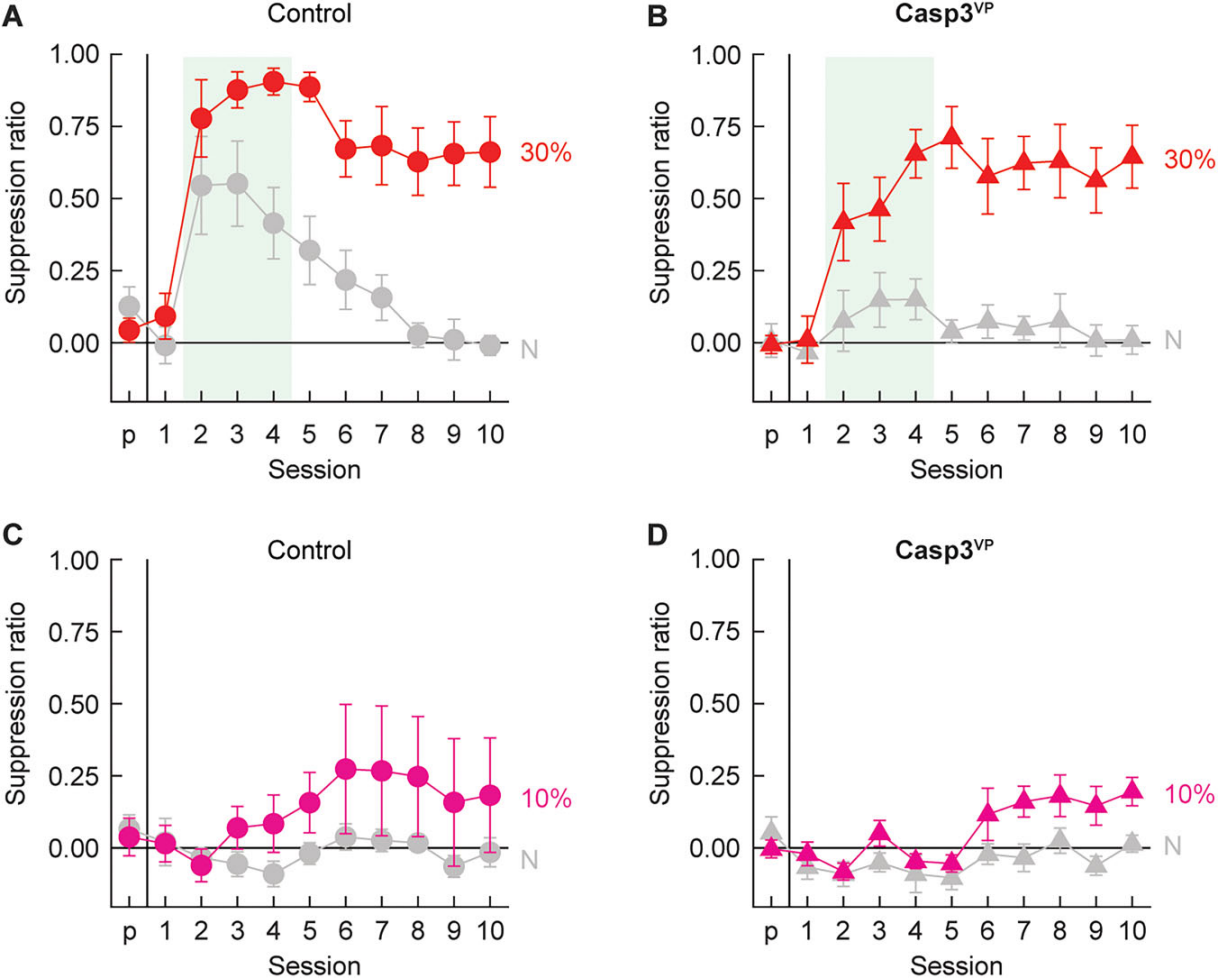
Control and Casp3^VP^ responding during minimal threat learning. Mean ± SEM suppression ratios for threat (red, *p *= 0.3, and pink, *p *= 0.1) and neutral (gray, *p *= 0) from pre-exposure (*p*) through the 10 minimal threat learning sessions (1–10) are shown for (***A***) control (circles) and (***B***) Casp3^VP^ rats (triangles) in the 30% probability condition and (***C***, ***D***) the 10% probability condition.

Over the 11 minimal threat learning sessions, rats acquired greater suppression to the threat cue, with greatest suppression observed to the 30% threat probability cue. Deleting ventral pallidum neurons had no impact on the emergence of discrimination in either probability condition (Fig. 6*A*–*D*) but abolished generalization of responding to the neutral cue in the 30% probability condition (Fig. 6*B*). Revealing expected patterns of discrimination across all rats, ANOVA for the suppression ratio [between-subject factors, group (Control^VP^ and Casp^VP^), sex (female and male), and probability (10 or 30%); within-subject factors, cue (threat vs neutral) and session (11, pre-exposure through session 10)] found a significant main effect of cue (*F*_(1,15) _= 87.83; *p *= 1.16 × 10^−7^) and significant interactions for cue × session, cue × probability, and cue × session × probability (all *F* > 3; all *p *< 0.01). Most critically, ANOVA revealed significant interactions of session × group (*F*_(10,150) _= 2.42; *p *= 0.011) and session × probability × group (*F*_(10,150) _= 2.17; *p *= 0.023). Casp3^VP^ rats in the 30% threat probability condition exhibited less overall suppression than controls. This decrease meant Casp3^VP^ rats never demonstrated suppression to the neutral cue above pre-exposure levels. Despite disrupting generalization, caspase-mediated ventral pallidum neuronal deletion did not disrupt discrimination. ANOVA revealed no significant interactions of cue × group (*F*_(1,15) _= 0.11; *p *= 0.75), cue × probability × group (*F*_(1,15) _= 0.33; *p *= 0.57), or cue × session × group (*F*_(10,150) _= 0.19; *p *= 0.997).

#### Influence of biological sex

ANOVA found no main effects of sex but a significant session × sex × probability (*F*_(10,150) _= 2.18; *p *= 0.022) interaction. Male rats in the 10% condition acquired higher suppression than females. This pattern was reversed in the 30% condition, with female rats acquiring slightly higher suppression than males.

#### Extinction test

**Figure 7.**
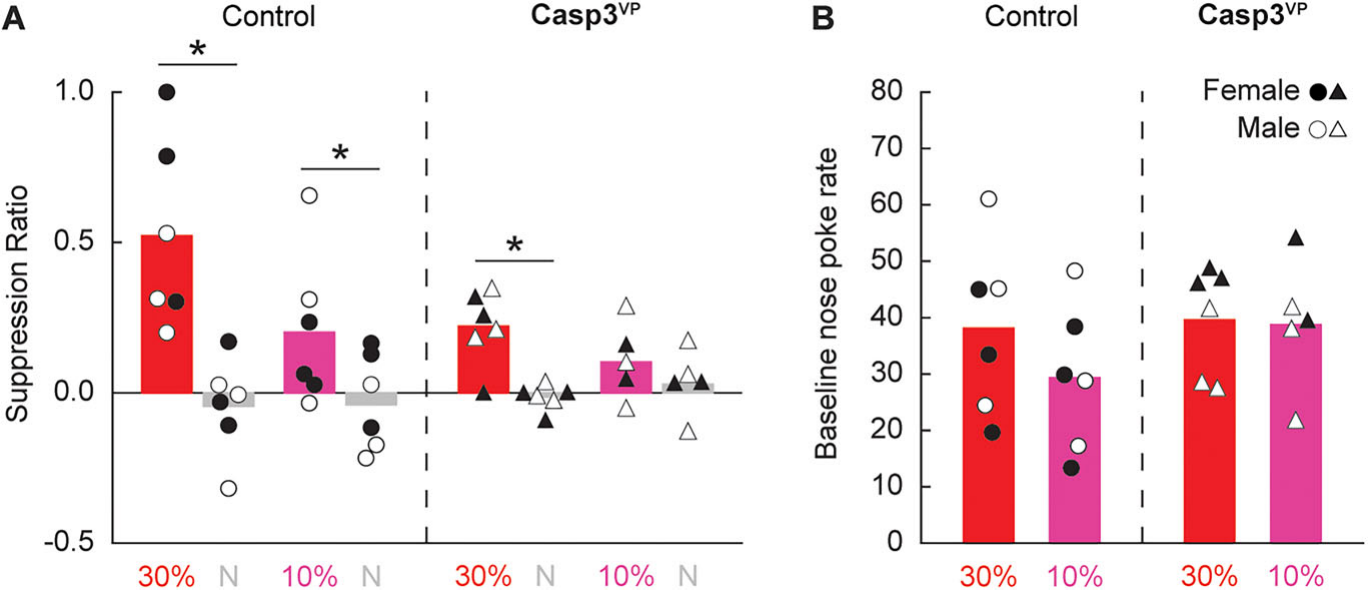
Control and Casp3^VP^ responding during extinction test. ***A***, Group Mean (bars) and individual (female, filled; male, open) suppression ratios for threat (red, *p *= 0.3, and pink, *p *= 0.1) and neutral (gray, *p *= 0) from the extinction test are shown control (left, circles) and Casp^VP^ rats (right, triangles). ***B***, Group Mean and individual baseline nose poke rates are shown (color and shape as in ***A***). *Independent samples *t* test, *p *< 0.05.

Extinction testing revealed discrimination and sensitivity to the threat probability manipulation but reduced responding to the threat cue in Casp^VP^ rats (Fig. 7*A*). Confirming discrimination, ANOVA [between-subject factors, group (Control^VP^ and Casp^VP^), sex (female and male), and probability (10 or 30%); within-subject factor, cue (threat vs neutral)] found a significant main effect of cue (*F*_(1,15) _= 24.76; *p *= 0.0001). The 95% BCIs confirmed differential responding to the threat and neutral cues by Control^VP^ rats (M, 0.40; 95% CI [0.20, 0.61]) and Casp^VP^ rats (M, 0.16; 95% CI [0.08, 0.23]). Revealing sensitivity to the threat probability manipulation, ANOVA found a significant probability × cue interaction (*F*_(1,15) _= 4.79; *p *= 0.045). Independent sample *t* test found greater responding to the 30% threat cue than the 10% threat cue across all rats (*t*_(21) _= 2.10; *p *= 0.047), while no significant difference was observed to the neutral cue (*t*_(21) _= 0.39; *p *= 0.70). Demonstrating reduced responding to the threat cue in Casp^VP^ rats, ANOVA found a significant cue × group interaction (*F*_(1,15) _= 5.01; *p *= 0.041). Independent sample *t* test comparing differential threat/neutral suppression by Control^VP^ and Casp^VP^ rats fell short of significance (*t*_(21) _= 2.02; *p *= 0.056). Group differences in suppression ratio could not be attributed to differences in baseline nose poking (Fig. 7*B*). All groups showed equivalent baseline nose poke rates during the extinction test (all *t* < 1.1; *p *> 0.6).

### Experiment 3

Experiment 2 revealed that ventral pallidum neuron deletion abolishes fear generalization and reduces threat responding during extinction. Experiment 3 examined possible roles of the nucleus accumbens input to the ventral pallidum in the generalization and expression of responding to the 30% threat cue.

#### Histology

**Figure 8.**
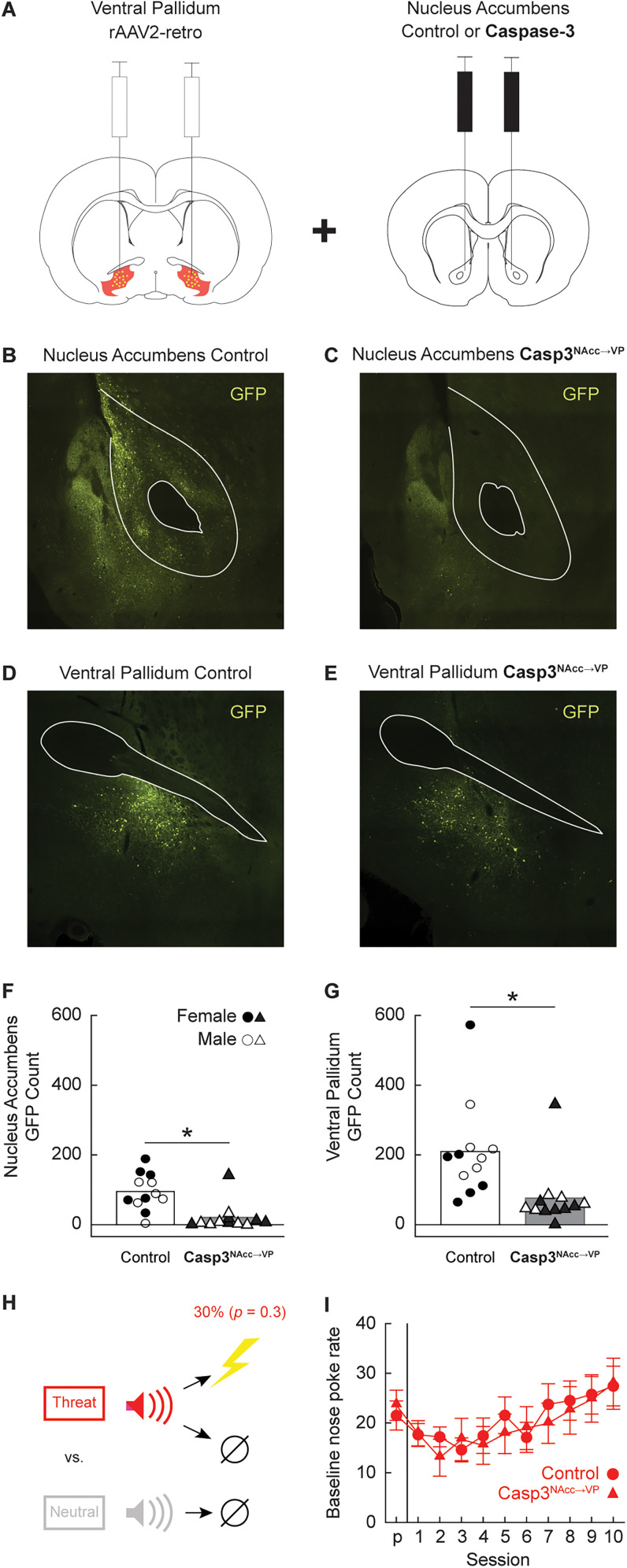
Histology, experimental design, and baseline nose poke rate. ***A***, All rats received bilateral rAAV2-retro infusions into the ventral pallidum (left). Control rats received mCherry infusions into the nucleus accumbens core, while Casp3^NAcc→VP^ rats received cre-dependent caspase-3 infusions into the nucleus accumbens core (right). Representative fluorescent images of the nucleus accumbens show GFP+ neurons in (***B***) controls but not in (***C***) Casp3^NAcc→VP^ rats. The accumbens core and anterior commissure are outlined. Representative fluorescent images of the ventral pallidum show GFP + neurons in (***D***) controls and in (***E***) Casp3^NAcc→VP^ rats. The anterior commissure is outlined. Group Mean (bars) and individual (female, filled; male, open) nucleus accumbens neuron number (***F***) and ventral pallidum neuron number (***G***) are shown for control (circles) and Casp3^VP^ (triangles). ***H***, Minimal threat learning procedure consisted of 10 s auditory cues predicting unique footshock probabilities: threat (red, *p *= 0.3) and neutral (gray, *p* = 0). ***I***, M ± SEM baseline nose poke rate from pre-exposure (*p*) through the 10 minimal threat learning sessions (1–10) is shown for control (circles) and Casp3^NAcc→VP^ rats (triangles). *Independent samples *t* test, *p *< 0.05.

Control^NAcc→VP^ rats received bilateral rAAV2-retro into the ventral pallidum and mCherry into the nucleus accumbens (Fig. 8*A*, left). Casp3^NAcc→VP^ rats also received bilateral rAAV2-retro into the ventral pallidum and, now, cre-dependent caspase-3 into the nucleus accumbens core (Fig. 8*A*, right). Subcommissural as well as accumbens core GFP fluorescence was bilaterally quantified at bregma 0.00 and at bregma +1.68 for each rat. Control^NAcc→VP^ rats (Fig. 8*B*,*D*) showed higher levels of GFP fluorescence in both accumbens and ventral pallidum sections. In contrast, there was an overall reduction in GFP fluorescence in both accumbens and ventral pallidum sections within Casp3^NAcc→VP^ rats (Fig. 8*C*,*E*). Independent sample *t* test found significantly higher nucleus accumbens GFP counts in Control^NAcc→VP^ (M, 94.83) compared with Casp3^NAcc→VP^ rats (M, 20.58; *t*_(22) _= 3.91; *p *= 0.001; Fig. 8*F*). The same pattern was found for the ventral pallidum (*t*_(22) _= 6.07; *p *= 8.03 × 10^−5^; Fig. 8*G*). GFP expression did not differ between females and males in either the accumbens core (*t*_(22) _= 1.09; *p *= 0.29) or the ventral pallidum (*t*_(22) _= 0.17; *p *= 0.87). Observing reduced ventral pallidum GFP was not expected. We speculate on the reason for this finding in the discussion.

#### Baseline nose poke rates

Following recovery each rat received 11 sessions of minimal threat learning (Fig. 8*H*). The baseline nose poke rate increased for all groups across the 11 sessions (Fig. 8*I*). Male rats poked at higher rates than female rats in both groups. ANOVA for the baseline nose poke rate [between-subjects factor, sex (female and male) and group (Control^NAcc→VP^ or Casp3^NAcc→VP^); within-subject factors, session (11, pre-exposure through Session 10)] found a main effect of session (*F* = 11.10; *p *= 5.35 × 10^−15^) and a main effect of sex (*F* = 11.45; *p *= 6.37 × 10^−10^).

#### Minimal threat learning

**Figure 9.**
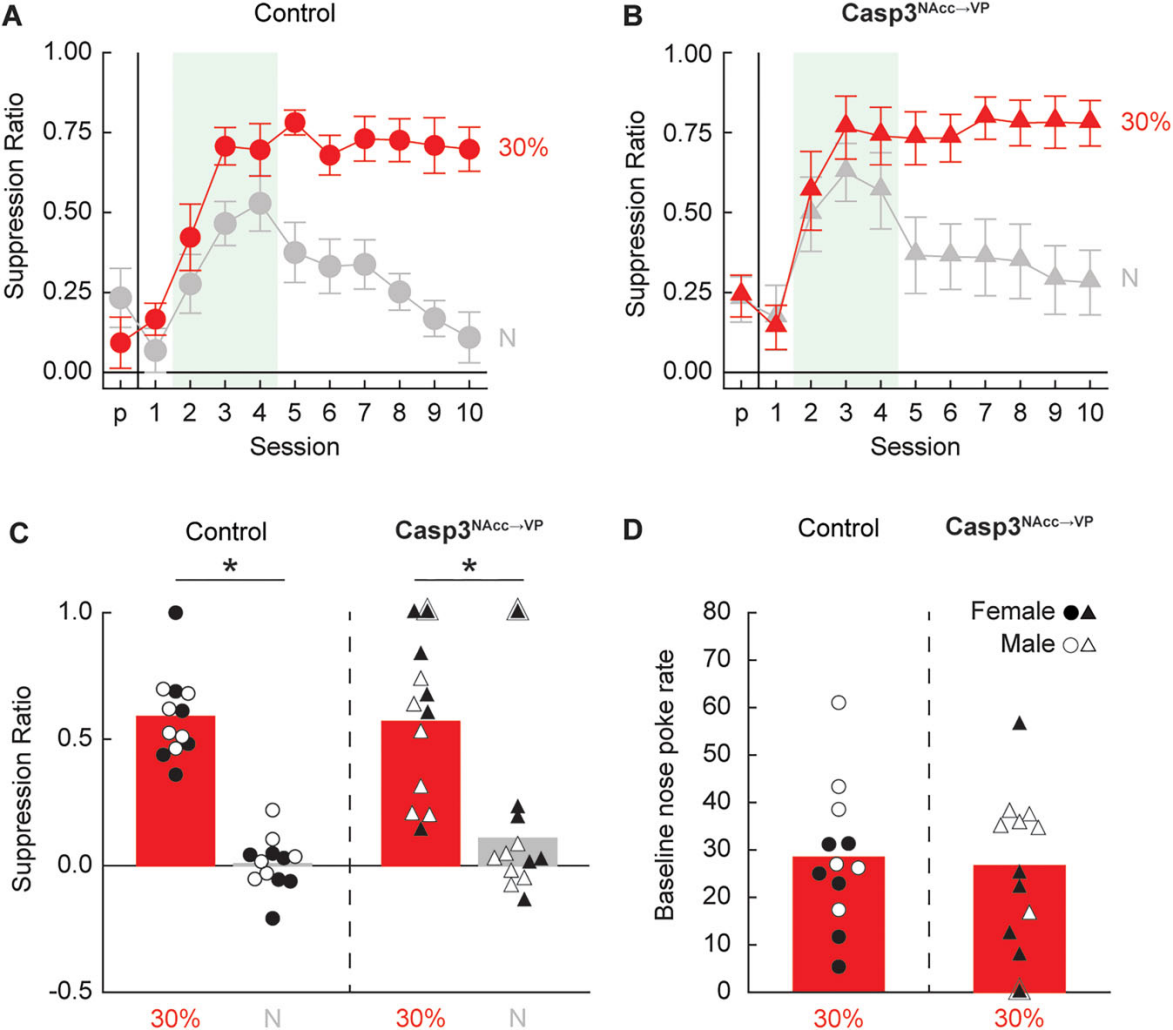
Control and Casp3^NAcc→VP^ responding during minimal threat learning and extinction. Mean ± SEM suppression ratios for threat (red, *p *= 0.3) and neutral (gray, *p *= 0) from pre-exposure (*p*) through the 10 minimal threat learning sessions (1–10) are shown for (***A***) control (circles) and (***B***) Casp3^NAcc→VP^ rats (triangles). ***C***, Group Mean (bars) and individual (female, filled; male, open) suppression ratios for threat (red, *p *= 0.3, and pink, *p *= 0.1) and neutral (gray, *p *= 0) from the extinction test are shown for control (left, circles) and Casp3^NAcc→VP^ rats (right, triangles). ***D***, Group Mean and individual baseline nose poke rates from the extinction test are shown for control and Casp3^NAcc→VP^ rats (color and shape as in ***D***). *Independent samples *t* test, *p *< 0.05. A single rat that did not poke during testing is outlined with an additional triangle in ***C*** and ***D***.

Deleting ventral pallidum-projecting accumbens neurons had no impact on discrimination or generalization of fear responding (Fig. 9*A*,*B*). ANOVA for suppression ratio [between-subject factors, sex (female and male) and group (Control^NAcc→VP^ or Casp3^NAcc→VP^); within-subjects factor, cue (threat or neutral) and session (11, pre-exposure through Session 10)] found a main effect of cue (*F* = 63.00; *p *= 1.32 × 10^−7^), a main effect of session (*F* = 18.79; *p *= 4.47 × 10^−24^), and a significant cue × session (*F* = 13.86; *p *= 1.89 × 10^−18^) interaction. No interactions or main effect of group were observed (all *F* < 1.40; *p *> 0.25).

#### Influence of biological sex

ANOVA found no main effects of sex but a significant cue × sex interaction (*F* = 6.32; *p *= 0.021). Female rats showed greater suppression to the neutral cue than male rats, overall.

#### Extinction test

Extinction testing revealed complete discrimination in all rats (Fig. 9*C*). ANOVA [between-subject factors, group (Control^NAcc→VP^ or Casp3^NAcc→VP^) and sex (female and male); within-subject factor, cue (threat vs neutral)] revealed a main effect of cue (*F*_(1,20) _= 102.78; *p *= 2.51 × 10^−9^). The 95% BCIs confirmed differential responding to the threat and neutral cues by Control^NAcc→VP^ rats (M, 0.58; 95% CI [0.49, 0.67]) and Casp^NAcc→VP^ rats (M, 0.46; 95% CI [0.30, 0.61]). All groups showed equivalent baseline nose poke rates (Fig. 9*D*) during the extinction test (*p *< 0.05).

## Discussion

By manipulating footshock probability, we uncovered a boundary (between 20 and 10%), below which fear discrimination occurs in the absence of fear generalization. Threat cues on either side of this boundary supported different levels of fear-related responding during an extinction test (30% = 20% > 10%). Using these minimal threat learning procedures, we demonstrated the roles of the ventral pallidum in generalizing fear-related responding to a neutral cue, and expressing responding to a threat cue. At the same time, we found that neither ventral pallidum roles depend on inputs from the nucleus accumbens.

Before discussing our results further, limitations and caveats must be pointed out. The ventral pallidum neuronal deletion was pan-neuronal and nearly complete. Almost no neurons remained in the ventral pallidum of deleted rats. Because the ventral pallidum contains GABAergic, cholinergic, and glutamatergic projection neurons, we cannot be certain which type(s) were responsible for which ventral pallidum roles in behavior. Furthermore, the ventral pallidum neuronal deletion was permanent. This means we cannot be certain if the changes in behavior during the extinction test resulted from the absence of ventral pallidum neurons “during” the test itself. Ventral pallidum neuronal deletion may have impacted initial learning, which then manifested in an extinction responding deficit. We feel that pan-neuronal deletion is justified. No prior study had examined such a role of the ventral pallidum. If permanently deleting all neurons had no impact on any aspect of behavior, it would have provided powerful negative evidence against the roles of the ventral pallidum.

A somewhat surprising finding was that ventral pallidum neuronal deletion did not reduce responding for food reward. This might have been anticipated given that manipulations of the ventral pallidum can reduce feeding and promote food aversion (Cromwell and Berridge, 1993; Smith et al., 2009). However, more recent work shows that chronic, chemogenetic inhibition “or” excitation of the ventral pallidum promotes weight gain in rats without altering feeding (Doucette et al., 2022). Chronic ventral pallidum manipulations may be resulting in compensatory mechanisms, accounting for discrepancies with the effects of acute manipulations.

We found that infusing rAAV2-retro in the ventral pallidum and cre-dependent caspase-3 into the nucleus accumbens reduced neuronal number in the ventral pallidum. Although this reduction was insufficient to alter fear generalization and expression, it was unexpected. Since this study, we have performed another study using the same AAV constructs to delete paraventricular thalamus inputs to the ventral pallidum (Moaddab et al., unpublished observations). That study found no evidence of ventral pallidum neuronal deletion. Furthermore, that study quantified the nucleus accumbens as a control region and further found no evidence of deletion. It is not a matter of the dual viral approach generally failing. We suspect that the close proximity of the nucleus accumbens to the adjacent lateral ventricle allowed some caspase-3 to migrate to the nearby ventral pallidum, resulting in a partial deletion.

The negative findings of Experiment 3 are perhaps even more compelling given that some ventral pallidum neurons were deleted in addition to complete deletion of ventral pallidum-projecting, nucleus accumbens neurons. Still, it is premature to conclude there is no role for this pathway in modulating fear responding. Heterogeneous nucleus accumbens neuron types project to the ventral pallidum, with D1-receptor and D2-receptor expressing populations being mostly unique (Kupchik et al., 2015). In mice, optogenetic activation of D2-ventral pallidum–projecting accumbens neurons, but not D1-expressing neurons, increased anxiety-like behavior in the elevated plus maze, light/dark test, and novelty suppression of feeding (Correia et al., 2023). Furthermore, our approach deleted core and shell accumbens neurons projecting to the ventral pallidum. Our negative finding may have been the result of manipulating all accumbens to ventral pallidum pathways, spanning cell types and region of origin.

What is the ventral pallidum doing in minimal threat learning settings? One hypothesis is that the ventral pallidum is necessary to fully process footshock. Partially supporting this hypothesis, the ventral pallidum plays a role in organizing behavioral responding to nociceptive events (Asgharieh-Ahari et al., 2023; Ji et al., 2023). Perhaps then, the ventral pallidum is also necessary to fully process nociceptive events, such as footshock. This might explain why we observed overall reductions in threat cue responding during extinction. However, if the ventral pallidum were necessary to fully process footshock, we would expect greater reductions in threat cue responding over the 10 minimal threat learning sessions. Specifically, responding to the 10% threat cue—which was already low in intact rats—should have been abolished in rats with ventral pallidum neuronal deletion. We did not observe this.

Perhaps the ventral pallidum is necessary to process footshock in order to strengthen cue–shock associations. This is generally consistent with reports showing ventral pallidum correlates of reward prediction error (Ottenheimer et al., 2020). This could explain why there was no specific deficit in threat cue responding during the 10 minimal threat learning sessions. The presence of footshock during the learning sessions reduced the need to retain and recall the cue–shock association. The absence of footshock during extinction testing required rats to recall the cue–shock association. Rats with ventral pallidum neuronal deletion may have shown reduced threat cue responding during extinction because they had formed weaker cue–shock associations that were more difficult to recall during extinction testing. In further support, extinction testing took place 72 h following the last learning session (a weekend break), making recall of prior learning more challenging.

Drawing from studies of credit assignment (Rushworth et al., 2011), the ventral pallidum may track trial history (Ottenheimer et al., 2020) for purposes of assigning received foot shocks to cues. This process could not be revealed by traditional pavlovian fear conditioning studies, because most studies employ a single cue and footshock. As a result, there is no question as to which cue the shock should be assigned to. We employed relatively short sessions that contained 20 total cue presentations (10 threats and 10 neutral). Our rats were tasked with determining to which cue the footshock should be assigned. By representing the chronological, cue presentation history prior to footshock receipt, rats would ensure that the shock was attributed to the most recent cues. However, maintaining a chronological, “multiple”-cue presentation history, and assigning some degree of shock to each prior cue would mean erroneously assigning footshock to the neutral cue. This is because neutral cue presentations will proceed threat cue presentations roughly half the time. If the ventral pallidum normally tracks cue presentation history and ventral pallidum neuronal deletion abolishes this ability, then rats would only attribute footshock to the most recent cue—which is always the threat cue. This is the exact pattern we observe.

It is likely that the ventral pallidum contributes to multiple threat processes. Though entirely speculative, it is possible that these distinct processes are tied to unique neuron types. For example, GABAergic ventral pallidum neurons may mediate responding to threat cues. This might be akin to roles of GABAergic ventral pallidum neurons in mediating risk-related decision–making (Farrell et al., 2021; Hernández-Jaramillo et al., 2023). Cholinergic ventral pallidum neurons may contribute to prediction error (Danielmeier et al., 2015) or track cue presentation history (Brzosko et al., 2017). These signals would normally be used to strengthen cue–shock associations and assign shock to preceding threat cues. Should these signals exist, they can only function through projections with partner brain regions.

The ventral pallidum is anatomically connected with a multitude of brain regions that contribute to fear learning and expression (Root et al., 2015; Ray et al., 2022). Most interesting, cholinergic and GABAergic ventral pallidum neurons project directly to the basal amygdala (Haber et al., 1985). The basolateral amygdala's essential role in learning and expression of fear responding (LeDoux et al., 1990; Goosens and Maren, 2001; Lee et al., 2001; Koo et al., 2004; McDannald and Galarce, 2011) makes these projections particularly compelling. GABAergic projections to the basolateral amygdala may promote threat responding, while cholinergic projections may strengthen cue–shock associations.

Here we show that minimal threat learning procedures can distinguish fear generalization from fear discrimination. We used these procedures to reveal a novel for the ventral pallidum in fear generalization. Near-future work will link genetic neuron types to specific threat processes (shock processing, prediction error, and trial history). This work will further reveal the neural circuits through which the ventral pallidum shapes threat responding. Our results and novel experimental approach are working toward a more complete description of the neural mechanisms for fear generalization. The goal of this research is to identify new neural targets for therapies to restore selective fear to individual with anxiety disorders.

## Synthesis

Reviewing Editor: Timothy Jarome, Virginia Polytechnic Institute and State University Decisions are customarily a result of the Reviewing Editor and the peer reviewers coming together and discussing their recommendations until a consensus is reached. When revisions are invited, a fact-based synthesis statement explaining their decision and outlining what is needed to prepare a revision will be listed below. The following reviewer(s) agreed to reveal their identity: Jocelyn Richard, Kyle Smith.

Two expert reviewers have assessed your manuscript. Both reviewers found your manuscript to be strong and of broad interest to readers of eNeuro. However, they recommended several edits including some additional viral confirmation/analysis, that you separate sexes in graphs to help illustrate specific effects and make several text edits, as outlined below.

Reviewer #1:

In this manuscript the authors assess the role of ventral pallidum neurons and inputs from the nucleus accumbens in fear discrimination and generalization. They first establish that a 30% footshock probability results in fear cue discrimination and generalization, while a 10% probability results in discrimination without generalization. Then they show that virally mediated deletion of cell bodies in the ventral pallidum (VP) reduces generalization under 30% probability conditions. Finally, they show that deletion of nucleus accumbens neurons projecting to the VP does now disrupt discrimination or generalization. Overall, this is a rigorous set of studies, with appropriate controls and analyses, and the manuscript is scholarly. I just have a few moderate concerns and/or suggestions.

Main Comments

1. Given the significant effects of sex, and interactions between sex and probability of shock, as well as sex and cue type, it would be useful to show the male and female data plotted separately, as well as together.

2. It isn't clear to me whether there is near complete neuronal loss in VP as the authors state, or whether there is just loss of neurons that are targeted by the retro-AAV in VP. Did the authors conduct any analysis of the remaining neurons other than examining GFP expression (such as with a NeuN stain)? If not, it seems possible that the retro-AAV may only target a subset of VP neurons (such as those with stronger intra-VP connectivity) and that the caspase may selectively delete this subset that expressed GFP in the controls. Verification of the full extent of neuronal loss in this tissue would be informative for interpreting the results. I would be surprised if there truly was general neuronal loss in VP, but no effect on baseline operant responding for food.

3. The authors document a decrease in GFP particles in VP after the deletion of NAc neurons projecting to VP, but it isn't clear whether this is driven by loss of cell bodies in VP, or loss of NAc terminals. Example images from VP in this experiment would be helpful for interpreting the effect of the projection-specific deletion.

Minor Comments

1. The display of the tables is distorted in the pdf

2. Stats are missing in this statement: "The same pattern was found for the ventral pallidum ([stats]; Figure 7E).

3. Line 70: "we sought to develop behavioral procedures that dissociate fear generalization from fear generalization" <- generalization is repeated

Reviewer #2:

In three experiments, the authors establish behavioral learning criteria under different probabilities of shock (assayed as reduced seeking of food), ablate the ventral pallidum to assess its role, and ablate ventral pallidum inputs from the nucleus accumbens to similarly assess the role of this pathway. Experiment 1 does a nice job establishing the behavioral trends. It is an exceptionally compelling setup for assessing generalization of cues in addition to the effect of threat cue effects themselves, and in being able to disentangle effects of threat-cue responses vs. general interest in pursuing food when brain manipulations are made. Experiment 2 shows a critical role for the ventral pallidum in fear generalization. Experiment 3 shows a lack of a role for the accumbens-pallidum pathway in this.

The manuscript is well written and clear, and the experiments are well justified. The discussion and interpretation of the results is generally excellent. The findings are quite interesting, and the authors should be applauded for the rigor and effort in generating them.

I have one concern, and several minor suggestions.

For experiment 2, Figure 4, as well as experiment 3, figure 7 - the example expression is nice. However, it is important to know the spatial extent of cell deletion across animals. For example, histology maps could be generated that combine animal expression data (either min/max expression plots or plots that have each animals' expression semi-transparently overlaid), as is common in histology plots that involve injection of substances. The reason is that there is usually some unintended viral spread outside of the target area, often dorsally up the injector tract. It is important to know if there was spread to other areas that was consistent across animals. In other words, data ought to be provided showing that cell deletion at the target site was the common effect across the group of animals, and that there was not common cell deletion in other unintended areas. I do not think the authors need to do cell counts in areas beyond the target sites, but I do think this issue should be attended to with histology mapping plots. As a side point as well, it seems like the accumbens medial shell was affected in addition to the core in experiment 3 example histology images; if that is accurate, it might warrant clarification in the manuscript.

MINOR:

As a suggestion, when there are sex differences, graphs separating sex could be helpful to convey the statistical findings.

Experiment 2 - In baseline, the ventral pallidum Casp3 group worked more for food, which I gather is female-driven. This is the opposite of the literature, in which inhibition or lesions of ventral pallidal neurons tend to suppress food seeking and food intake. I don't doubt the data, but it is a curious finding. It is tangential to their main goal here, but a discussion sentence or two might be worthwhile since reward related functions are discussed in the introduction.

I found it hard to decipher the figures in relation to the figure legends. I would suggest additional language in the legends. For example, I gather Fig 1 shows training prior to the introduction of shock conditions, but if one just looks at this figure going from panel B to C, the impression is that C reflects the shock conditions rather than pre-shock training. This is just a minor suggestion. It took me a bit of time to figure out what this figure was conveying.

Why does the neutral cue have a non-zero suppression ratio for 30% and 20% conditions, but not 10%? I would find an explanation helpful. I am also curious why the neural cue is around 0 during the extinction test in relation to the prior days.

The authors could explain why they used a VI schedule for pellet delivery. As a theoretical point, which the authors can tend to or ignore at their discretion, a VI reward schedule cerates a situation in which animals seek food that comes at, presumably, surprising times. Thus, the animals are seeking food under conditions of uncertainty. Suppression of this food seeking by shock cues would seem to be hard to interpret in relation to the internal model of what is going to happen in the task. Perhaps animals are weighing probabilities - for example, food will occur at some point vs. x% chance of a shock. The other scenario is that food will always predictably occur, if delivered on an FR schedule, and animals will then weigh that probability vs. the chance of a footshock. In short, the food seeking suppression is clear and compelling. But, I would imagine a fair amount of (unknown) probability analysis of cost-benefit is occurring in these conditions that bias the animals' choices. One might find different results under an FR schedule of reward, for example.

With a myriad of sex differences discovered, readers might benefit from a brief discussion on this and what, if any, relation it has to the literature and/or human sex differences related to fear. This is up to the authors.

The accumbens-pallidum pathway is a clearly justified choice for studying. However, the introduction mentions other connections, emphasizing the BLA for example. This might be revisited in the discussion more thoroughly - if not the accumbens, then what?

## Author Response

Computational Neuroscience Model Code Accessibility Comments for Author (Required):

N/A Synthesis Statement for Author (Required):

Two expert reviewers have assessed your manuscript. Both reviewers found your manuscript to be strong and of broad interest to readers of eNeuro. However, they recommended several edits including some additional viral confirmation/analysis, that you separate sexes in graphs to help illustrate specific effects and make several text edits, as outlined below.

Reviewer #1:

In this manuscript the authors assess the role of ventral pallidum neurons and inputs from the nucleus accumbens in fear discrimination and generalization. They first establish that a 30% footshock probability results in fear cue discrimination and generalization, while a 10% probability results in discrimination without generalization. Then they show that virally mediated deletion of cell bodies in the ventral pallidum (VP) reduces generalization under 30% probability conditions. Finally, they show that deletion of nucleus accumbens neurons projecting to the VP does now disrupt discrimination or generalization. Overall, this is a rigorous set of studies, with appropriate controls and analyses, and the manuscript is scholarly. I just have a few moderate concerns and/or suggestions.

Main Comments 1. Given the significant effects of sex, and interactions between sex and probability of shock, as well as sex and cue type, it would be useful to show the male and female data plotted separately, as well as together.

We have included sex in the plots showing individuals, with the females being filled markers and the males open. The reviewer is correct that sex interactions were observed in Experiment 1. However, we did not observe significant sex interactions for our main findings of Experiments 2 and 3. For this reason we do not plot all data separately throughout the manuscript.

2. It isn't clear to me whether there is near complete neuronal loss in VP as the authors state, or whether there is just loss of neurons that are targeted by the retro-AAV in VP. Did the authors conduct any analysis of the remaining neurons other than examining GFP expression (such as with a NeuN stain)? If not, it seems possible that the retro-AAV may only target a subset of VP neurons (such as those with stronger intra-VP connectivity) and that the caspase may selectively delete this subset that expressed GFP in the controls. Verification of the full extent of neuronal loss in this tissue would be informative for interpreting the results. I would be surprised if there truly was general neuronal loss in VP, but no effect on baseline operant responding for food.

We have now mapped neuronal deletion in the ventral pallidum as well as provided representative intact and deleted images. We observe - as far as we can see - complete neuronal loss where deletions occurred. This was not surprising to us, as the AAV-retro construct uses a general promoter that should be taken up by most all neuron types. We suspect mostly GABA neurons are deleted. When we infused cre-dependent caspase into the ventral pallidum of Gad1-cre rats we see a very similar pattern of deletion. Immunohistochemistry is not good at detecting Gad1. RNAscope is required. We did not perform RNAscope verification of Gad1 deletion in this study because we did not have a neuron-type specific manipulation.

We are also surprised by the lack of an effect of ventral pallidum neuronal deletion on baseline nose poking. We now discuss this after the results. [Lines 421-427] 3. The authors document a decrease in GFP particles in VP after the deletion of NAc neurons projecting to VP, but it isn't clear whether this is driven by loss of cell bodies in VP, or loss of NAc terminals. Example images from VP in this experiment would be helpful for interpreting the effect of the projection-specific deletion.

The revised manuscript includes example images [Figure 8]. You bring up a good point. We definitely observe neurons in the ventral pallidum of casp3 rats. There may have been some loss of fibers contributing to the decreased fluorescence.

Minor Comments 1. The display of the tables is distorted in the pdf We apologize. The table got distorted in the conversion from an excel file to a pdf. The table is clear in the revised manuscript.

2. Stats are missing in this statement: "The same pattern was found for the ventral pallidum ([stats]; Figure 7E).

The t-statistic and corresponding p-value has been added [Lines 372-373].

3. Line 70: "we sought to develop behavioral procedures that dissociate fear generalization from fear generalization" <- generalization is repeated Fixed! - Reviewer #2:

In three experiments, the authors establish behavioral learning criteria under different probabilities of shock (assayed as reduced seeking of food), ablate the ventral pallidum to assess its role, and ablate ventral pallidum inputs from the nucleus accumbens to similarly assess the role of this pathway. Experiment 1 does a nice job establishing the behavioral trends. It is an exceptionally compelling setup for assessing generalization of cues in addition to the effect of threat cue effects themselves, and in being able to disentangle effects of threat-cue responses vs. general interest in pursuing food when brain manipulations are made. Experiment 2 shows a critical role for the ventral pallidum in fear generalization. Experiment 3 shows a lack of a role for the accumbens-pallidum pathway in this.

The manuscript is well written and clear, and the experiments are well justified. The discussion and interpretation of the results is generally excellent. The findings are quite interesting, and the authors should be applauded for the rigor and effort in generating them.

I have one concern, and several minor suggestions.

For experiment 2, Figure 4, as well as experiment 3, figure 7 - the example expression is nice. However, it is important to know the spatial extent of cell deletion across animals. For example, histology maps could be generated that combine animal expression data (either min/max expression plots or plots that have each animals' expression semi-transparently overlaid), as is common in histology plots that involve injection of substances. The reason is that there is usually some unintended viral spread outside of the target area, often dorsally up the injector tract. It is important to know if there was spread to other areas that was consistent across animals. In other words, data ought to be provided showing that cell deletion at the target site was the common effect across the group of animals, and that there was not common cell deletion in other unintended areas. I do not think the authors need to do cell counts in areas beyond the target sites, but I do think this issue should be attended to with histology mapping plots. As a side point as well, it seems like the accumbens medial shell was affected in addition to the core in experiment 3 example histology images; if that is accurate, it might warrant clarification in the manuscript.

Reviewer 1 raised the same points. The revised manuscript includes a complete mapping of YFP expression and neuronal deletion for Experiment 2. Both YFP expression and neuronal deletion were specific to the ventral pallidum in the anterior and middle sections (bregma 0.00) but there was some spread outside of the boundaries in the posterior sections. This is when the anterior commissure opens, and we see some dorsal spread. In our description of the pathway findings, we have been careful to not overstate the specificity of the pathway manipulation to the accumbens core.

To the medial shell observation. We agree with the reviewer. Throughout the manuscript we are careful to avoid stating our manipulation was specific to the core. And we discuss the possibility that our negative result was due to targeting both the core and the shell. [Lines 446-448] MINOR:

As a suggestion, when there are sex differences, graphs separating sex could be helpful to convey the statistical findings.

Bar plots with individual data points are now broken down by sex.

Experiment 2 - In baseline, the ventral pallidum Casp3 group worked more for food, which I gather is female-driven. This is the opposite of the literature, in which inhibition or lesions of ventral pallidal neurons tend to suppress food seeking and food intake. I don't doubt the data, but it is a curious finding. It is tangential to their main goal here, but a discussion sentence or two might be worthwhile since reward related functions are discussed in the introduction.

We agree and have now added a paragraph to the discussion pointing out this perhaps unexpected finding. [Lines 420-427] I found it hard to decipher the figures in relation to the figure legends. I would suggest additional language in the legends. For example, I gather Fig 1 shows training prior to the introduction of shock conditions, but if one just looks at this figure going from panel B to C, the impression is that C reflects the shock conditions rather than pre-shock training. This is just a minor suggestion. It took me a bit of time to figure out what this figure was conveying.

We have extensively revised the figures and the figure legends. But yes, Figure 1C shows baseline nose poke rates during the cue pre-exposure and shock sessions. We felt it was important to show because baseline nose poke rate is used to calculate suppression ratio.

Why does the neutral cue have a non-zero suppression ratio for 30% and 20% conditions, but not 10%? I would find an explanation helpful. I am also curious why the neural cue is around 0 during the extinction test in relation to the prior days.

The answer is that auditory cues by themselves have a small ability to interfere with nose poking. It is better to compare the final suppression ratio level of the neutral cue to initial pre-exposure level. All three groups show non-zero levels of neutral cue suppression during pre-exposure. By the final day of minimal threat learning, suppression levels are similar or lower to these pre-exposure levels. We also find some experiment to experiment variability in the absolute levels of suppression shown. Sometimes we do see zero suppression by experiments' end. But even when we don't see that, we most always see final levels of neutral cue suppression similar to or lower than pre-exposure levels.

The authors could explain why they used a VI schedule for pellet delivery. As a theoretical point, which the authors can tend to or ignore at their discretion, a VI reward schedule cerates a situation in which animals seek food that comes at, presumably, surprising times. Thus, the animals are seeking food under conditions of uncertainty. Suppression of this food seeking by shock cues would seem to be hard to interpret in relation to the internal model of what is going to happen in the task. Perhaps animals are weighing probabilities - for example, food will occur at some point vs. x% chance of a shock. The other scenario is that food will always predictably occur, if delivered on an FR schedule, and animals will then weigh that probability vs. the chance of a footshock. In short, the food seeking suppression is clear and compelling. But, I would imagine a fair amount of (unknown) probability analysis of cost-benefit is occurring in these conditions that bias the animals' choices. One might find different results under an FR schedule of reward, for example.

Practically, a variable interval schedule encourages consistent nose poke rates across the entire session. A ratio schedule might see rats poking a lot in the beginning, getting full, then tapering off towards the end. We want stable responding across the session to have consistent suppression ratio measures. That said, we agree with your overall point of rats making cost-benefit type computations in this setting. A fixed ratio would eliminate any uncertainty as to when food is delivered, likely altering the rats decision to poke or not. We now provide a statement in the methods about why we chose a variable schedule [Lines 145-147].

With a myriad of sex differences discovered, readers might benefit from a brief discussion on this and what, if any, relation it has to the literature and/or human sex differences related to fear. This is up to the authors.

Yes, so we intentionally designed our experiments and analyzed our data to reveal possible sex-specific contributions of the ventral pallidum. In some cases, we found significant interactions with sex. However, the main findings of the manuscript - reduced fear generalization and reduced fear expression - are observed across both sexes. Further, neither the female nor the male rats required accumbens inputs to the ventral pallidum for these effects. Because the main findings did not differ by sex, we chose to de-emphasize discussion of sex.

The accumbens-pallidum pathway is a clearly justified choice for studying. However, the introduction mentions other connections, emphasizing the BLA for example. This might be revisited in the discussion more thoroughly - if not the accumbens, then what? A good point. Right now, we are thinking more about the likely outputs of the ventral pallidum that are mediating these fear effects. The basolateral amygdala is a prime candidate. We discuss this possibility. [Lines 494-501]
