# Supplementary figures and images for: Ventral Pallidum Neurons Are Necessary to Generalize and Express Fear-Related Responding in a Minimal Threat Setting
Source: eNeuro. 2024 Nov 26;11(11):ENEURO.0124-24.2024. doi: 10.1523/ENEURO.0124-24.2024 (PMC11595600; doi:10.1523/ENEURO.0124-24.2024)

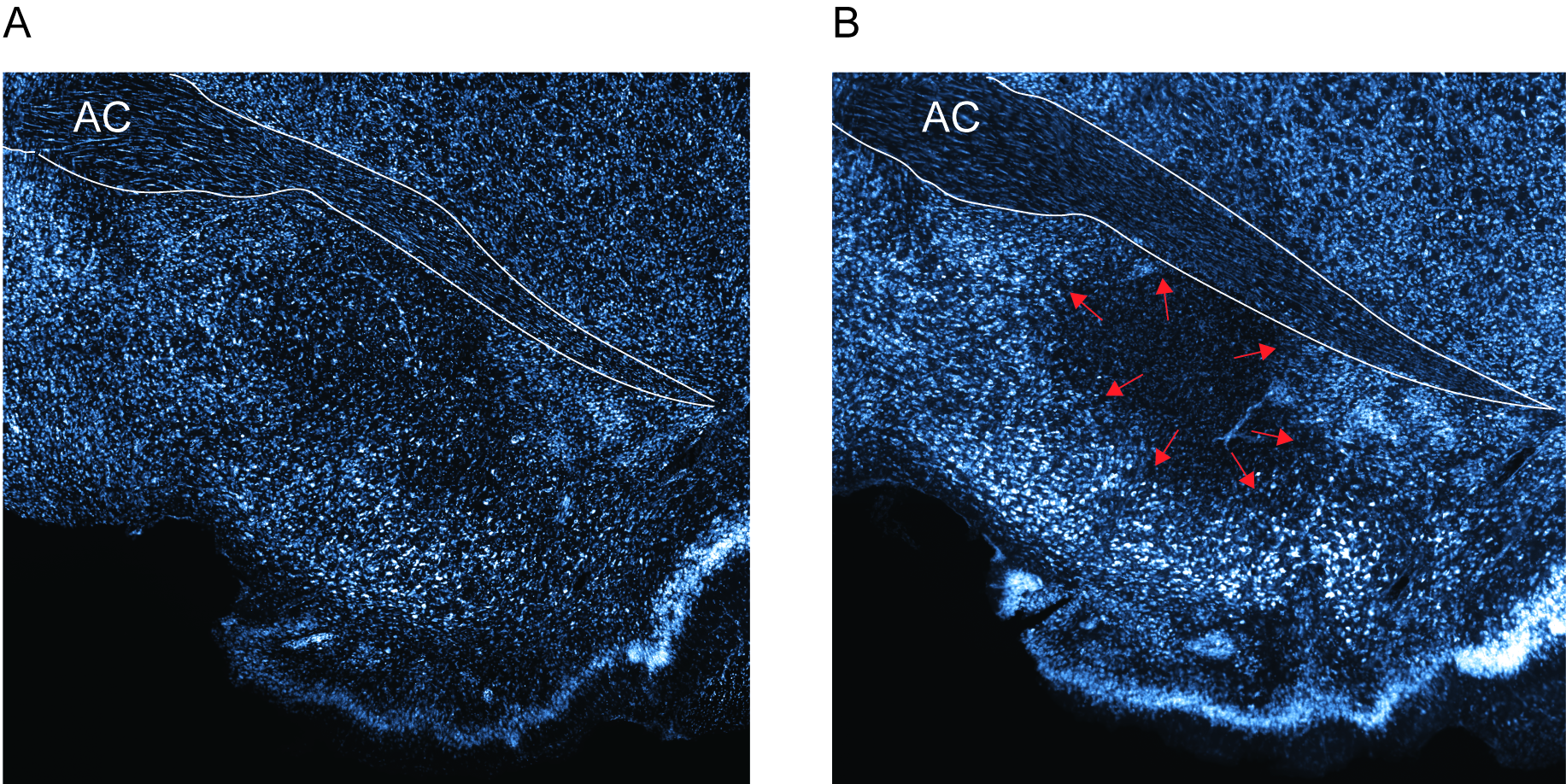

Supplement: Figure 4-1 — Representative NeuroTrace Images from Experiment 2 (A) A coronal section from a control rat containing the ventral pallidum is stained with NeuroTrace. The anterior commissure (AC) is outline in white. Sub-commissural ventral pallidum neurons are intact. (B) A coronal section from a caspase-3 rat containing the ventral pallidum is stained with NeuroTrace. The anterior commissure (AC) is outlined in white. Sub-commissural ventral pallidum neurons are deleted with little or no evidence of remaining neurons. Download Figure 4-1, TIF file. [file eneuro-11-ENEURO.0124-24.2024-s002.tif]
